# Beyond Raynaud's: Atypical Peripheral Vascular Manifestations in a Case of CREST Syndrome

**DOI:** 10.1002/ccr3.71815

**Published:** 2026-01-10

**Authors:** Sakshi Kumari, Fathimathul Henna, Umama Alam, Fazia Khattak, Kamil Ahmad Kamil

**Affiliations:** ^1^ Shimoga Institute of Medical Sciences Shimoga India; ^2^ Dubai Medical College for Girls Dubai UAE; ^3^ Khyber Medical College Peshawar Pakistan; ^4^ Internal Medicine Department Mirwais Regional Hospital Kandahar Afghanistan

**Keywords:** amputation, crest syndrome, ischemia, Raynaud's phenomenon, sclerosis

## Abstract

Peripheral vascular disease (PVD) is a rare but severe symptom of CREST syndrome, which itself is a limited cutaneous sclerosis. Even though Raynaud's phenomenon is the gold standard of CREST, the development of critical limb ischemia and self‐amputation is rare. We report a case of a 48‐year‐old female with CREST syndrome who presented with progressive ischemia in both upper and lower limbs, leading to spontaneous auto‐amputation of multiple fingers/toes. The patient exhibited sclerodactyly, dry gangrene, delayed capillary refill, and tactile deficits in peripheral pulses. ANA and anti‐Scl 70 antibody were positive. CT angiography demonstrated segmental and progressive obstruction of multifocal peripheral arteries. The patient was operated on for amputation of necrotic digits and was initiated on immunomodulatory therapy. Following treatment, monitoring revealed stabilization without further progression. This case highlights the possible seriousness of vascular complications in CREST syndrome. Prompt diagnosis and treatment are necessary to prevent irreversible ischemic damage.

## Key Clinical Message

CREST syndrome, a limited systemic sclerosis variant, may rarely present with progressive peripheral artery disease causing critical limb ischemia and auto‐amputation. Absence of traditional cardiovascular risk factors should raise suspicion for autoimmune vasculopathy. Early recognition, vascular imaging, immunomodulatory therapy, and multidisciplinary management are essential to prevent irreversible complications and limb loss.

## 1 Introduction

Peripheral vascular disease (PVD) is a rare and progressive circulation disorder. It is more commonly seen in lower limbs when compared to upper limbs. A rare autoimmune disorder like CREST syndrome can cause narrowing of vessel lumen, accelerated atherosclerosis, and vascular inflammation, resulting in PVD. CREST syndrome is a localized variant of systemic sclerosis, characterized by calcinosis (Calcium skin deposits) and Raynaud's phenomenon. The prevalence is relatively low with 253–286 cases per million [1]. With a 4:1 ratio, women are at a larger risk than men. PVD in CREST syndrome ranges from Raynaud's phenomenon to gangrene.

## 2 Case Presentation

**FIGURE 1:**
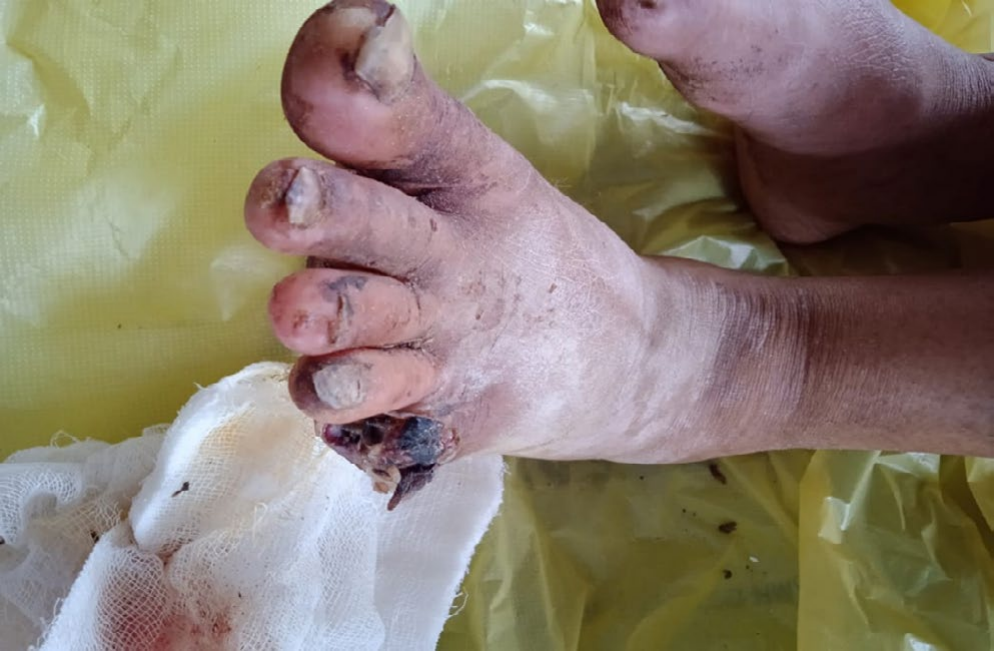
Blackish discoloration of the right and left lower limbs.

A female patient, aged 48 years, a homemaker by occupation, was admitted to the emergency department. Her chief complaints were blackish discolouration of fingers of upper limb since 1 year associated with loss of sensations and claudication of upper limb at rest. This was followed by auto‐amputation of both right and left index and little fingers. The patient experienced bilateral foot pain for 2 months described as an aching pain with claudication at rest. History of blackish discoloration of right and left feet which involved the toes of both feet (Figure 1), followed by auto‐amputation of 5th toe of left foot was also present. This was associated with loss of sensation and claudication of lower limb at rest. There was no history of trauma.

There was a history of the patient's fingers turning pale on exposure to cold and stress which was associated with tingling and numbness. There was swelling of digits and joint stiffness which was not associated with pain. There were complaints of difficulty in swallowing and weight loss. There was a history of dry skin especially in the areas of forearms and lower legs. Additionally, she experienced dry skin, especially in the areas of the forearms and lower legs. There was no history of syncope, blackouts, transient loss of consciousness, fainting, blurring of vision. There was no history of chest pain, abdominal pain, or alterations in bladder and bowel movements.

There was no history of similar complaints in the past. The patient was not a known case of diabetes, hypertension, coronary artery disease (CAD), tuberculosis, or asthma. Her family history was insignificant.

## 3 Examination of Patient

On general physical examination under proper exposure of light, the patient was moderately built, nourished, and well oriented in time, place, and person. The blood pressure was 110/60 mm hg, pulse 100 beats/min, respiration 16 beats/min, and temperature 36.8°C. There was no pallor, icterus, cyanosis, clubbing, pedal edema, or lymphadenopathy.

The patient was explained about the procedure and after taking consent, was locally examined in a well lit room in supine position. The limbs of the patients were hanging down the bed, the 5th left toe and index and little fingers of both hands were amputated. There was sclerodactyly of the digits and black mummified gangrene of the remaining toes and fingers associated with loss of sensations. The upper limb was cold below the elbow joint and the lower limbs were cold below the knee joint. The nails showed transferred ridges and were brittle. Tenderness was present at the junction of the gangrenous area and normal skin. The skin appeared to be shiny, dry and the joints were stiff. The fingers turned pale from exposure to cold. The right and left dorsalis pedis, anterior tibial, posterior tibial, brachial, and ulnar arteries were not palpable; however, both radial arteries were feeble.

CVS, GI, RS, and CNS were found to be normal on systemic examination.

Peripheral pulses were feeble or absent distally; both radial arteries were weakly palpable, and dorsalis pedis and posterior tibial pulses were absent.

### 3.1 Palpation and Examination of Lower Limbs

**FIGURE 2:**
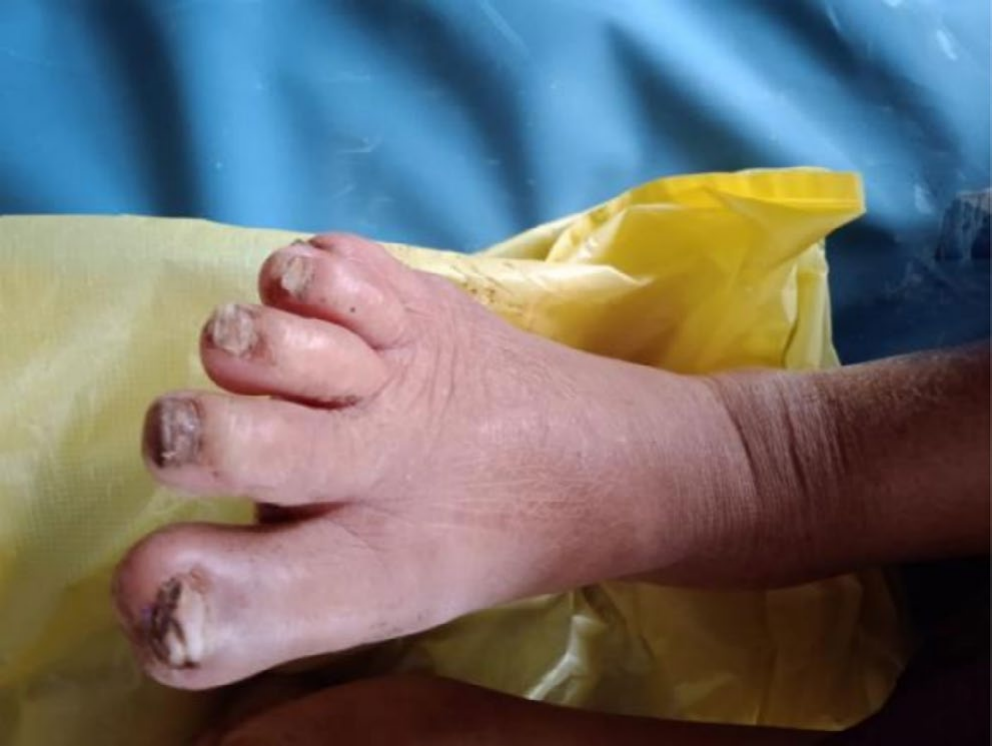
Sclerodactyly of the toes with thickened skin and gangrenous changes.

Movements of joints: At intertarsal, midtarsal, and ankle joints, movements were lost. At knee and hip, movement was possible. Muscle tone and power were normal while bulk of muscle was reduced. No signs of ischemia were noted while toes turned pale on exposure to cold. Sclerodactyly of the toes was noted with thickened skin and marked pallor noticed at 45 degrees on Buerger's test (Figure 2). Capillary and venous filling time were prolonged and numbness of toes was present.

### 3.2 Palpation and Examination of Upper Limbs

**FIGURE 3:**
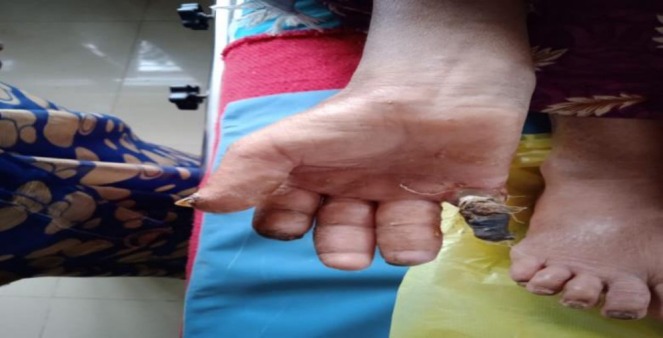
Amputation of index and little fingers with swelling and skin thickening. Black mummified appearance with a clear line of demarcation on both hands.

Upon examination of hands, placed on knees in a sitting position, dryness of skin was observed. Index and little fingers were both amputated with swelling in fingers and thickened skin over fingers (Figure 3). Black mummified appearance of right and left index and little fingers with a clear line of demarcation was observed. No engorged veins were observed; however, capillary filling time and venous filling time were prolonged. Palpation revealed both hands to be cold with yellowish discoloration of the skin. Sclerodactyly of the digits and stiffness at the joints was present. Black mummified appearance, dry gangrene of right and left index and little fingers, loss of sensations with a clear mark of demarcation was noted. The gangrenous fingers were hard and shriveled up, and tenderness was noted at the junction of the gangrenous area and normal tissues.

Upon sensory examination of the limbs, crude and fine touch, pain, and temperature sensations above the wrist were normal. Biceps and triceps reflexes were present on both sides while the supinator reflex could not be elicited. Regional lymph node examination was normal. Motor examination revealed normal tone and power but decreased bulk of muscles (Table 1). On palpation, movements of joints at the intercarpal and wrist joints were lost. At the shoulder and elbow, movement was possible.

### 3.3 Circumference Measurement

**TABLE 1:** Circumference measurement of the upper limbs.

	Right	Left
Forearm	22	25
Arm	18	21

### 3.4 Differential Diagnosis

The differential diagnoses based on patient examination were atherosclerosis, Polyarteritis nodosa, SLE, and Raynaud's disease.

## 4 Investigation, Management and Treatment

CBC, RBS, LFT, RFT, Lipid profile, ANA Anti‐Scl 70 antibody, serum electrolytes, ABPI, RF, CRP, PT, FBS, CT Angiography Peripheral arms and legs, X‐ray were ordered for the patient. The significant findings were as follows:

- ANA‐ Positive
- Anti‐Scl 70 antibody‐ 56 AU/ML (negative)
- RF‐ Normal
- Anticentromere antibodies: Positive
- CT Angiography of Peripheral arms:
  Right: Aberrant origin of right subclavian artery with selective and complete blockade of radial artery with progressive blockade of other peripheral arteries.
  Left: Vessels showed biphasic flow and impending ischemia.
- X ray of chest: Normal.

Given the absence of renal or pulmonary involvement and no clinical symptoms indicative of ANCA‐associated vasculitis, ANCA testing was not necessary. This corresponds with the typical serological profile of limited cutaneous systemic sclerosis (CREST syndrome), in which anticentromere antibodies are usually positive, anti‐Scl‐70 antibodies are negative, and ANCA testing is not routinely indicated unless systemic vasculitis is suspected.

The patient was initially managed with vasodilators, consisting of nifedipine, along with aspirin and pentoxifylline to enhance peripheral blood flow. Supportive care comprised pain management, wound care, and protection from cold exposure. Despite these measures, ischemia worsened, confirmed by CT angiography demonstrating complete occlusion of the radial artery and progressive distal arterial involvement.

Given the severity and irreversibility of ischemia, amputation of the affected digits was done to prevent further necrosis and systemic complications. Postoperatively, the patient was started on immunomodulatory therapy, comprising low‐dose corticosteroids and methotrexate under rheumatology supervision. Regular follow‐up was suggested to monitor for additional systemic manifestations of limited systemic sclerosis.

## 5 Discussion

Peripheral Vascular Disease (PVD) is characterized by significant narrowing of the arteries distal to the aortic arch and presents with intermittent claudication and acute or critical limb ischemia [2]. The patient presented with claudication of both upper and lower limbs, accompanied by ischemia in the digits. Thus, the presentation of the patient was typical of PVD. However, it has been found to be more prevalent in male patients above 60, leading us to question why a female who would not be typically at risk of being affected by it, developed it? Smoking, diabetes and a family history of PVD have shown to be significant factors towards developing PVD in comparatively younger adults [3]. The patient was not exposed to any of these factors, making the case peculiar. Amputation is required in only 4% of the patients with PVD, placing this patient in an even smaller group amongst the very few people who suffer from this condition [4]. Moreover, peripheral vascular disease is an independent predictor of CVS abnormalities in 50% of these patients, which on systemic examination were found absent [5]. The patient was also classified as a case of Raynaud's syndrome, which causes characteristic color changes in the digits as a result of vasospasm, especially in cold weather [6]. However, it was not determined if the Raynaud's disease developed as a result of PVD or if the PVD developed as a result of her Raynaud's disease; as both have a cause and effect relationship [7]. It can be said that she developed secondary Raynaud's syndrome based on the severity of her symptoms as secondary Raynaud's syndrome usually develops after 40 years of age and presents with severe symptoms that do not resolve on their own [7]. Her symptoms of dryness and discoloration of skin were typical of PVD, resulting from a reduced blood flow due to constriction and blockage of the arteries [8]. Furthermore, her weight loss can be explained with reference to the pain associated with her condition, causing a decreased appetite [9]. What is however surprising is the fact that weight loss has been proven to mitigate the symptoms of PVD, by reducing the stress on the cardiovascular system [10]. Whether this weight loss benefitted the patient or not, can only be determined by her BMI, which was normal, indicating that obesity was not the reason for her CVS being strained. Auto‐amputation was reported for this patient, indicating that her disease had progressed enough for the gangrenous digit to shrivel up and detach on its own. Swelling and edema of digits was also observed in this patient, a classic symptom of PVD due to inflammation of surrounding soft tissue [11].

In contrast to prior reported cases of CREST syndrome‐related peripheral vascular disease, the present case is distinct due to its bilateral symmetrical involvement of both upper and lower limbs and worsening of the condition to auto‐amputation. Most studies describe Raynaud's phenomenon and digital ulcers as the chief vascular symptoms, with lower limb affection being rare. For instance, Herrick et al. showed that digital ischemia in systemic sclerosis mainly involves the hands, with severe ischemia or gangrene being rare and usually confined to distal digits [12]. Similarly, a case series by Wigley and Flavahan highlighted that while vasospasm is frequent in systemic sclerosis, severe ischemic complications needing amputation are not common and generally unilateral [13]. In contrast, our patient presented with ischemic changes surpassing the digits, with complete radial artery obstruction and digital auto‐amputation in both hands and feet, underscoring a more severe and aggressive vasculopathic process. Moreover, unlike other cases where early immunosuppressive therapy restricted vascular progression, the late diagnosis and late manifestation in this patient likely give rise to irreversible tissue loss [14]. These differences highlight the heterogeneity and variability in vascular involvement in CREST syndrome and the need for high vigilance in atypical or fast progressive cases.

## 6 Conclusion

PVD in CREST syndrome is a rare but severe complication leading to progressive ischemia and auto‐amputation. This case is especially rare and unique due to its bilateral upper and lower limb manifestation, highlighting a more severe vascular manifestation than normally reported. This emphasizes the need for prompt recognition of atypical vascular features, especially in patients without conventional cardiovascular risk factors. Elevated clinical suspicion, thorough serological evaluation, and prompt immunomodulatory therapy are necessary to enhance outcomes and prevent irreversible complications.

## Author Contributions

**Kamil Ahmad Kamil:** supervision, writing – review and editing. **Sakshi Kumari:** conceptualization, data curation, writing – original draft. **Fathimathul Henna:** writing – original draft. **Umama Alam:** writing – original draft. **Fazia Khattak:** writing – review and editing.

## Funding

The authors have nothing to report.

## Ethics Statement

Ethical approval was not required for this case report.

## Consent

Written informed consent was obtained from the patient for the publication of this case report.

## Conflicts of Interest

The authors declare no conflicts of interest.

## Data Availability

All data generated or analyzed during this study are included in this published article.
